# Melatonin's protective effect against placental transfer of Methadone in mice: An experimental study

**DOI:** 10.18502/ijrm.v22i7.16957

**Published:** 2024-09-12

**Authors:** Maryam Akbarzadeh, Ramin Ataee, Farkhondeh Nemati, Abbas Ali Dehpouri, Fatemeh Shaki

**Affiliations:** ^1^Department of Biology, Faculty of Basic Sciences, Islamic Azad University, Qaemshahr Branch, Qaemshahr, Iran.; ^2^Medicinal Plants Research Center, Mazandaran University of Medical Sciences, Sari, Iran.; ^3^Pharmaceutical Sciences Research Center, Mazandaran University of Medical Sciences, Sari, Iran.

**Keywords:** Oxidative stress, Methadone, Melatonin, Pregnancy.

## Abstract

**Background:**

Methadone is a substance widely used in the substitution treatment of opiate addiction in pregnancy. The placental transfer of methadone influences oxidative stress processes. Melatonin is a hormone with antioxidant activity.

**Objective:**

This study aimed to evaluate the protective effects of melatonin on oxidative stress induced by the transfer of transplacental methadone in mice.

**Materials and Methods:**

In this experimental study, 36 female mice (2 months old, 20 ± 2 gr) were divided into 6 groups (n = 6/each) of control, methadone (0.3 mg/kg intraperitoneal, single dose) and melatonin (2, 4, and 6 mg/kg/day gavage) were administered 30 min before methadone, and one group received melatonin alone (0.6 mg/kg with single injection). Administration for 10 consecutive days of the pregnancy period was done. After baby mice were born, all neonatal mice were killed by beheading or sacrificing after anesthesia. The liver tissues were extracted. The samples were then sent for studying oxidative stress markers such as lipid peroxidation, glutathione, and protein carbonyl contents. Also, we have used the immunohistochemistry method for apoptotic markers such as: BAX, Bcl2, and Caspase3 for assaying apoptosis.

**Results:**

This study has shown that methadone caused a significant decrease in glutathione concentration (p = 0.035). Also, we observed a significant increase in lipid peroxidation and protein carbonyl contents (p = 0.015, 0.025 respectively). However, melatonin treatment significantly inhibited oxidative stress markers (p = 0.025). Also, apoptosis assay has shown that melatonin could decrease BAX and Caspase 9 as apoptotic and increase Bcl2 as an antiapoptotic proteins (p = 0.015).

**Conclusion:**

Our findings have shown that melatonin has a protective effect against oxidative stress and apoptosis induced by the placental transfer of methadone via its antioxidant effects.

## 1. Introduction

Methadone in the form of methadone hydrochloride is a synthetic opioid, with a chemical structure and function similar to morphine. It is used to relieve acute and chronic pain (1–5).

Methadone is also used as a low-dose drug with high oral bioavailability and long half-life as a substitute for the treatment of people addicted to heroin and other opioids. The bioavailability of methadone is 80% and its half-life is about 24 hr (1). Daily oral methadone dose of 60–100 mg prevents opioid withdrawal syndrome in opioid-dependent patients (6).

Methadone has a long half-life in tissues such as the liver and other organs due to its lipophilic properties. About 85–90% of plasma is bound to α-1-acid glycoprotein (7).

The use of methadone was recommended as the preferred maintenance treatment to eliminate opioid dependence during pregnancy (8). Methadone is found in breast milk, saliva, umbilical cord plasma, and amniotic fluid (9). Babies lack an effective blood-brain barrier for opioids. Therefore, opioid analgesics easily cross the placenta (9, 10).

Methadone as a synthetic opioid is commonly used in medication-assisted treatment program for distinguished patients, with addiction to other prescription or illicit opioids. Apart from its usage in opioid disorder therapy, it is used as an analgesic for severe pain management (7). Methadone as a unique µ-opioid receptor agonist has some pharmacological effects that might be mediated through the blockade of the N-methyl-D-aspartate receptor (11, 12).

The ability of methadone to control opioid addiction is due to several factors: reduction of physical and psychological effects of quitting (13), inhibition or reduction of desire (14), and reduction of intoxication caused by other drugs (13). Currently, the rising prevalence of methadone therapy exists in pregnant women with opioid use disorder. Most women who use methadone during pregnancy need to consider major risks to their infants because of placental transfer (15). Pregnancy age and P-glycoprotein expression are effective in the transmission of methadone from mother to fetus (14). Methadone induces receptor endocytosis, reduces opioid tolerance, and changes redox activity (15).

There are different views on the toxicity and side effects of drug delivery from mother to child and its effect on oxidative stress processes. Reactive oxygen species produced under pathological and physiological conditions in mammalian tissues can attack cell macromolecules and react with lipids, proteins, DNA, and cell damage (16). The physiology of the human has several mechanisms to inhibit the effects of these reactive species by producing antioxidant enzymes like glutathione and catalase. Antioxidants as exogenous agents can also play an important role in reducing the damage caused by free radicals (17).

Melatonin (N-acetyl-5-methoxytryptamine) as a hormone is produced by the pineal gland each night and participates in some important physiological functions such as circadian rhythms and general sleep patterns, reproductive system, and immunity. Melatonin also acts as an antioxidant to defend cells against free radical species, and oxidative stress also excites and promotes growth of the antioxidant enzymes' expression as superoxide dismutase, glutathione peroxidase, and catalase (18–21).

In this study, the protective effect of melatonin as an antioxidant on the parameters of oxidative stress induced by placental methadone transferred from mother to infant mice is investigated. The preprint version of this paper has already been uploaded on Research Square website: doi.org/10.21203/rs.3.rs-2722787/v1.

## 2. Materials and Methods

### Materials

This study is an experimental study. Darupakhsh Co. provided Methadone as a syrup (25 mg/5 ml) from the Drugs Control Center, Mazandaran University of Medical Sciences, Sari, Iran; Melatonin powder was purchased from Sigma Co. (Germany); Thiobarbituric acid, N-butanol, Tetrametoxypropane, Ellman's reagent (5,5 ' -dithiobis-2-nitrobenzoic acid, [DTNB]), 2,4-dinitrophenylhydrazine, all provided from Merck Co., Germany.

Primary anti-mouse antibodies (anti-bax antibody [B-9]: sc-7480, anti-Bcl-2 antibody [C-2] sc-7382, anti-caspase-3 antibody [E-8]: sc-7272), normal goat serum Immunoglobulin G (IgG), secondary anti-mouse antibody, m-IgGk, Biotin-Hydroperoxidase: sc-516102 all purchased from Santa-Cruz Co. USA. Diaminobenzidine, tween 80, hematoxylin provided from Sigma Co. (Germany), absolute alcohol, xylol, phosphate buffer powder, hydrogen peroxide, bovine serum albumin, Canada balsam, citric acid, sodium citrate and sodium chloride obtained from Merck Co. (Germany), ethyl alcohol, immunohistochemistry (IHC) super plus slides and cover slips provided from Kimia Gostar Co., Iran.

### Animal breading

36 Swiss albino mice (2 months old, 20 ± 2 gr) were obtained from Laboratory Animals Research Center, Mazandaran University of Medical Sciences, Sari, Iran and were stored in an air-conditioned environment with temperature of 22 ± 2 C, maintained at a 12 hr light/dark cycle, and free access to food and water.

1 male and 3 female mice were then put together in a cage and mated, both male and female mice were sexually mature at 8 wk and were able to give birth. The vulva of the female mouse was observed, and when it became swollen and red (this shows that the sexual cycle is at the proestrus point), she was chosen for mating. The female vulva was observed, and if the vaginal plug was detected, female mice whose vaginal plug had been confirmed were kept together in one cage, and treatments began.

### Sample size

a) mean of the outcome variable in group 1 = M1

b) mean of the outcome variable in group 2 = M2

c) SD of the outcome variable in group 1 = σ 1

d) SD of the outcome variable in group 2 = σ 2

The formula used in the calculation of sample size is given below: 


$$n=\frac{{\left({O1}^{2}+{O2}^{2}\right).\left[Z_{1-\alpha /2}+Z_{1-\beta }\right]}^{2}}{{(M_{1}-M_{2})}^{2}}$$

### Experimental design

Randomly, 36 mice were divided into 6 groups with 6 animals in each of the control group (normal saline), methadone group (0.3 mg/kg intraperitoneally, single dose), and different doses of melatonin group (2, 4, and 6 mg/kg/day gavages) were used (Table I). Melatonin was given 30 min before methadone. The study continued for over 10 days (the 8 ${\;}^{\text{th}}$ -18 ${\;}^{\text{th}}$ day of pregnancy), and treatments given were as a single injection for all groups. After the mice gave birth, the newborn mice were killed by beheading (animals were sacrificed after anesthesia by ketamine/xylazine with a ratio V/V [3/1]); after killing, the mice were buried hygienically and buried underground. These samples were considered for measuring the markers of oxidative stress such as glutathione (GSH) concentration, lipid peroxidation (LPO), and protein carbonyl (PrC). For assaying apoptosis, we used the IHC method for BAX, Bcl2, and Caspase3 protein expression. Animals were obtained from the Laboratory Animals Research Center, Mazandaran University of Medical Sciences, Sari, Iran.

### Measurement of LPO

Malondialdehyde (MDA) level, an index of LPO, was determined using Zhang et al.,2008 method (22). For which we first add 0.25 ml sulfuric acid (0.05 mol/l) in 0.2 ml samples (1 mg protein/ml), followed by 0.3 ml of 0.2% Thiobarbituric acid. The samples were then put in a warm water bath at 80 C for 30 min. In the next step, the micro-tubes were handed over to an ice bath, after which 0.4 ml n-butanol was added to each tube and moved forcefully. Following this, a centrifuge at 3500 × g for 10 min was done. Finally, the MDA amount was assayed by determining the absorbance of the supernatant at 532 nm with an enzyme-linked immunosorbent assay reader (ELX800, Biotek, USA). In this experiment, tetrametoxypropane as a standard was used and MDA concentration was shown as nmol/mg protein (22).

### Measurement of GSH content 

GSH content was assayed by the Ellman method (19), and DTNB as an indicator was used through the spectrophotometer method. In summary, 0.1 ml of tissue was put into 0.1 mol/l of buffer phosphate with 0.04% DTNB as a total volume of 3.0 ml (pH = 7.4), which is shown in yellow color and can be read by a spectrophotometer at 412 nm. The content as μg/mg protein has been shown (23).

### Measurement of PrC

PrC is produced as a result of the metal-catalyzed oxidation of amino acids such as lysine, and proline, while arginine and threonine remain as a result of the production of carbonylated forms. Thus, the PrC levels considered as a protein oxidation marker PrC were evaluated by spectrophotometric method with minor changes by Barreiro E and Zinellu E methods (24, 25). In summary, 200 μL of homogenate was extracted in 500 μl of 20% (w/v) maintained at 4 C for 15 min and treated with 500 μl of 0.2% 2,4-dinitrophenylhydrazine and 500 μl of 2 mol L-1 hydrochloric acid (HCl) as control group. The samples were then maintained at 37 for 1 hr with vortexing at 5-min intervals. In the end, the proteins were precipitated by adding 55 μl of 100% trichloroacetic acid. Then, samples were centrifuged and washed 3 times with 1000 μl of ethanol-ethyl acetate mixture and dissolved in 200 μl of 6 mol L-1 guanidine HCl. Finally, carbonyl content was determined at 365 nm wavelength.

### IHC

IHC procedure for assaying the localization of apoptotic proteins (BAX, Bcl2, Caspase9) in tissues was done based on routine protocols and according to our previous study (26). For this, first neonatal liver tissues were blocked in formalin 10%, after which tissue sections (3 µm) from paraffin blocks were prepared, then deparaffinized and hydrated. For antigen retrieval, the sections were put in citrate buffer (10 mM, pH = 6.0), and heated twice in the microwave at 750 V for 5 min. After a cool recycling period, they were washed 3 times in (tween buffer saline) and then incubated with normal goat IgG (10%) for 20 min. The sections were treated with 0.05% hydrogen peroxide and 10% BSA for 20 min to block the activity of endogenous peroxidase and non-specific proteins. Then, non-specific antigens were blocked by incubating sections in blocking buffer (TBS containing 10% goat serum, and 0.1% Triton X-100) for 20 min at room temperature. The final sections were incubated for 24 hr at 4 C with the specific IHC primary antibody (anti-Bcl2, anti-Caspase9, and anti-Caspase9, all in concentration 1:250) diluted in TBS containing 0.1% Triton X-100 in humidified chambers.

The sections were then washed with TBS 3 times and incubated with the secondary antibody (Donkey anti-goat IgG-HRP Conjugated) for 2 hr at 37 C. They were then incubated with the immunodetection solution (Diaminobenzidine + H ${\;}_{2}$ O ${\;}_{2}$ 0.05%) for 10 min, and washed with tap water. Finally, the slides were counterstained with hematoxylin. Parallel sections were processed as above to exclude non-specific staining, but specific primary antibodies were denied. Then, sections were observed under a light microscope at x100 or x400 magnification and scanned and examined by computer.

For assessing the immunoreactivity level, a semiquantitative method with microscopic study of slides for 4 grades (0, 1+, 2+, 3+) was used in which immunoreactivity, concerning the strength and width of distribution of precipitated dyes in nuclear and cytoplasm of the cells were considered.

**Table 1:** Treatments groups


**Group **	**Treatment**	**Route of administration**	**Duration of treatment**
**1**	2 mg melatonin + 0.3 mg/kg methadone	Melatonin (gavage), Methadone (IP)	10 days
**2**	4 mg melatonin + 0.3 mg/kg methadone	Melatonin (gavage), Methadone (IP)	10 days
**3**	6 mg melatonin + 0.3 mg/kg methadone	Melatonin (gavage), Methadone (IP)	10 days
**4**	6 mg melatonin (alone)	gavage	10 days
**5**	Methadone (0.3 mg/kg)	IP	10 days
**6**	Normal saline (0.9%)	IP	10 days
IP: Intraperitoneally			

### Ethical considerations

All the experiments were done based on NIH guidelines for laboratory animals of the Animal Ethical Committee of Deputy of Research, Mazandaran University of Medical Sciences, Sari, Iran (Code: IR.MAZUMS.REC.1398.1180). It is confirmed that all methods are reported in accordance with ARRIVE guidelines.

### Statistical analysis

All results were presented as mean ± SD, the statistical analyses were done using SPSS software, version 21, and assays were in triplicate. Also, statistical significance was determined using the one-way (ANOVA) test, followed by the post-hoc Tukey test, and was set at (p < 0.05). For IHC results, we used the Exact Fisher's test for qualitative data analysis (p < 0.05).

## 3. Results

### Effects of melatonin on LPO

**Figure 1:**
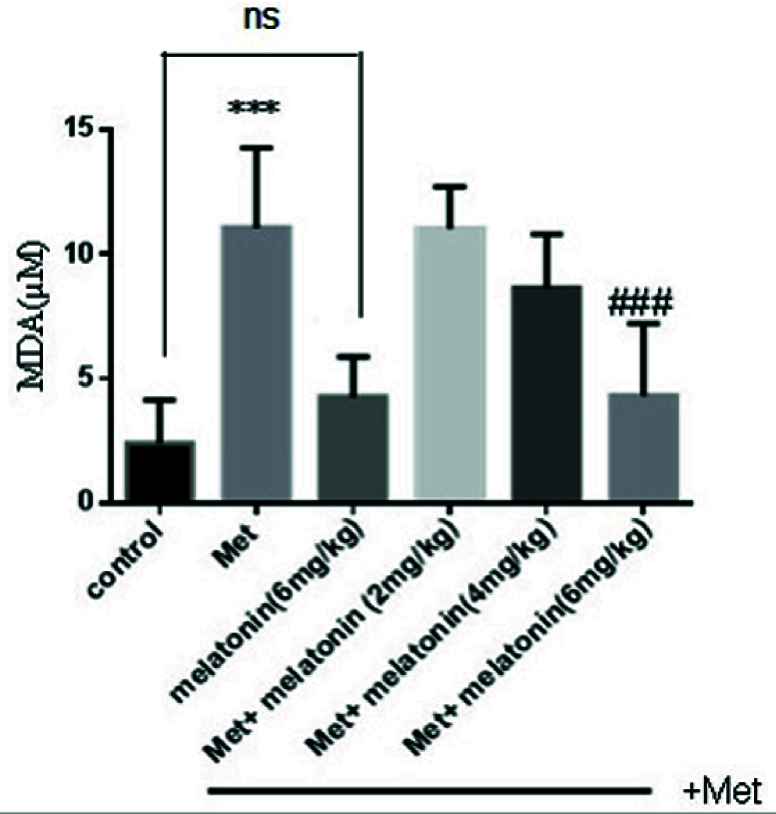
Effect of melatonin on Methadone-induced lipid peroxidation on liver tissues of neonate mice. Using thiobarbituric acid reagent. ***P < 0.001 compared with control mice, ###P < 0.001 compared with Met mice. Met: Methadone, MDA: Malondialdehyde.

MDA is a major product of LPO, and MDA elevation is known as an important marker for oxidative stress. Methadone administration enhanced MDA levels significantly (p < 0.001) as compared to the control group. As indicated, treatment with a high dose of melatonin (6 mg/kg) caused a significant decrease in LPO as compared with methadone treated group (p < 0.001), but melatonin in low and medium doses was without any significant effect on the LPO level (Figure 1).

### Effects of melatonin on GSH concentration

**Figure 2:**
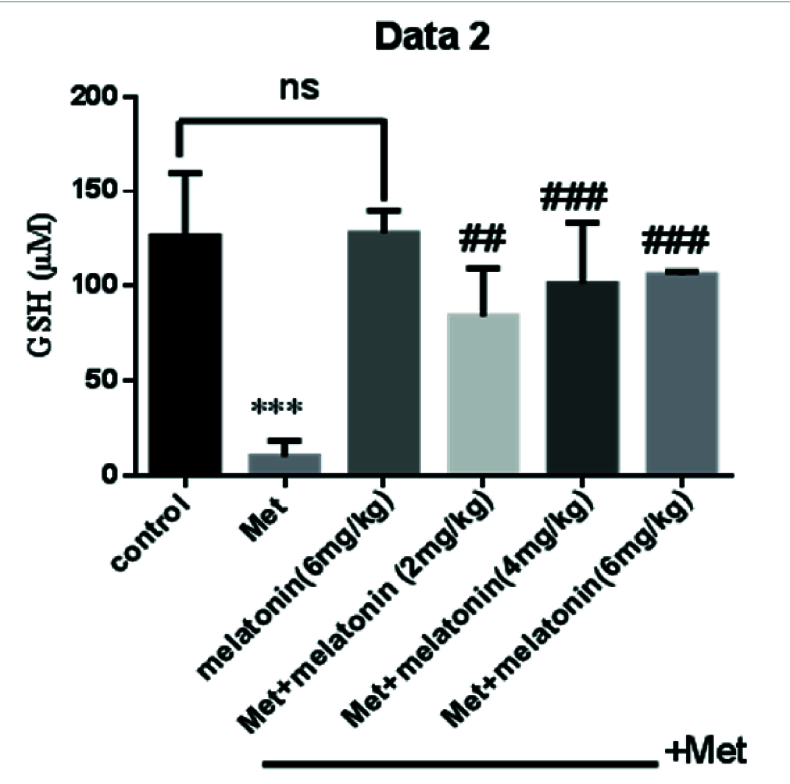
Effect of melatonin on GSH concentration on liver tissues of neonate mice. GSH concentration was measured using DTNB. ***P < 0.001 compared with control mice, ##P < 0.01, ###P < 0.001 compared with Met mice. Met: Methadone, GSH: Glutathione.

Methadone administration led to the significant depletion of GSH level (p < 0.001) as compared with the control group. Interestingly treatment with all 3 melatonin doses caused a significant increase in the level of GSH compared with the methadone group (p ≤ 0.001), respectively, for 3 doses, p < 0.001 (Figure 2).

### Effects of melatonin on PrC

**Figure 3:**
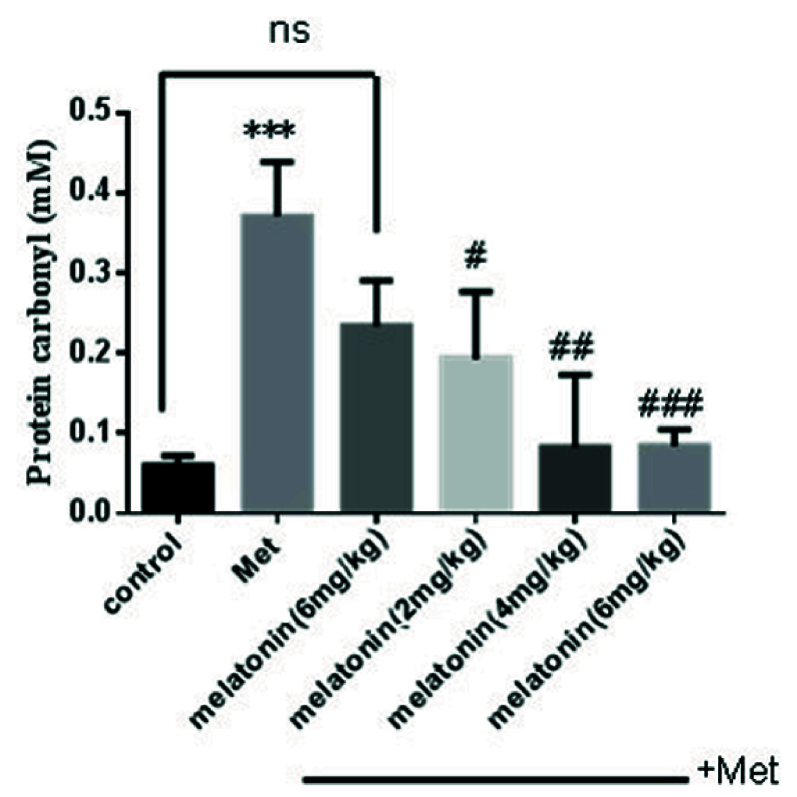
Effect of melatonin on protein carbonyl on liver tissues of neonate mice. ***P < 0.001 compared with control mice, #P < 0.05, ##P < 0.01, ###P < 0.001 compared with Met mice. Met: Methadone

A significant elevation of PrC has been observed in the methadone group compared with the control group (p < 0.001). Treatment with melatonin with different doses for 10 days had a significant effect on decreasing these oxidations at 3 doses of melatonin, 2 mg/kg (p = 0.002), 4 mg/kg (p = 0.008), and 6 mg/kg (p < 0.001) compared with methadone treated group (Figure 3).

### IHC results

**Figure 4:**
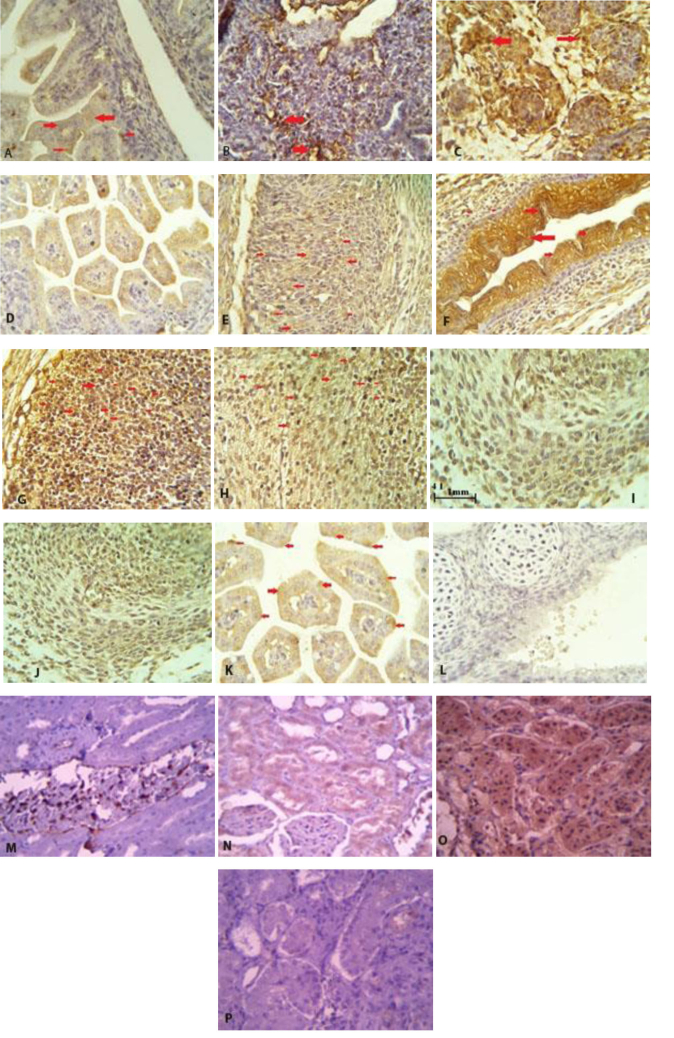
Immunohistochemistry results of apoptotic protein expression (BAX, BCl2, Caspase9) (X100) in liver tissues of neonate mice in different concentrations of melatonin (2, 4, 6 mg), Bax: Apoptotic factor, Melat: Melatonin, Met: Methadone, Bcl2: Anti-apoptotic factor. A) Bax expression, Melat 6 mg/kg/Met 10 mg/kg, grade: +, B) Bax expression, Melat 4 mg/kg/Met 10 mg/kg, grade: ++, C) Bax expression, Melat 2 mg/kg/Met 10 mg/kg, grade: +++, D) Caspase9 expression, Melat 6 mg/kg/Met 10 mg/kg, grade: +, E) Caspase9 expression, Melat 4 mg/kg/Met 10 mg/kg, grade: ++, F) Caspase9 expression, Melat 2 mg/kg/Met 10 mg/kg, Ggrade: +++, G) Bcl2 expression, Melat 6 mg/kg/Met 10 mg/kg, grade: +++, H) Bcl2 expression, Melat 4 mg/kg/Met 10 mg/kg, grade: ++, I) Bcl2 expression, Melat 2 mg/kg/Met 10 mg/kg, grade: +, J) Bcl2 expression with melatonin alone 4 mg/kg+, K) Caspase expression with melatonin alone 4 mg/kg+, L) Bax expression with melatonin alone 4 mg/kg+, M) Positive control for Bcl2, N) Positive control for caspase, O) Positive control for BAX, P) Negative control.

**Figure 5:**
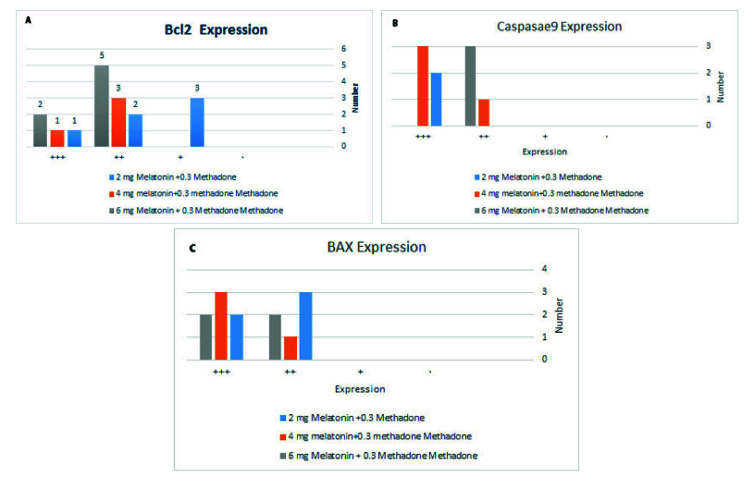
Immunohistochemistry results of apoptotic protein expression (BAX, BCl2, Caspase9) in liver tissues of neonate mouse in different concentrations of melatonin (2, 4, 6 mg) with Fisher's exact test. Decreased expression of apoptotic protein BAX at dose 6 mg/kg melatonin compared with dose 4 mg/kg and 2 mg/kg p > 0.05, and increased expression of anti-apoptotic protein Bcl2 at dose 6 mg/kg melatonin compared with 4 mg/kg and 2 mg/kg and decreased expression of apoptotic protein Caspase9 at dose 6 mg/kg compared with 4 mg/kg as it is noticeable but not significant p > 0.05, A) Bcl2 comparison curve, B) Caspase 9 comparison curve, C) Bax comparison curve.

In figures 4 and 5, the results of IHC in different concentrations of melatonin have been shown in the liver tissues of newborn mice. As shown, melatonin, almost in a dose manner, could attenuate the expression of apoptotic protein BAX and Caspase9 and increase anti-apoptotic protein expression, Bcl2. As BAX expression at a dose of 6 mg/kg, melatonin was decreased compared with the dose of 4 mg/kg and 2 mg/kg (Figure 4a, against 4b and 4c), (4d against 4e and 4f). Also, an anti-apoptotic protein, Bcl2 expression at a dose of 6 mg/kg melatonin compared with the dose of 4 mg/kg and 2 mg/kg has increased (Figure 4g against 4h and 4I).

## 4. Discussion

This study has shown the protective effect of melatonin against methadone administration in pregnant mice in their neonates. As we know, methadone is used as a weak opioid agonist for controlling withdrawal signs in addicts, and it is possibly used in addicted mothers, so the researchers are finding the route to ameliorate its adverse effects on the fetus. This study has been designed according to this background, considering the potent antioxidant properties of melatonin.

According to our results, methadone administration in mice for 10 consecutive days in the pregnancy period caused oxidative damage. Evaluation of oxidant biomarkers in newborn mice showed that methadone significantly increased LPO (the decadence) of lipid caused by the deletion of electrons from the cell membrane by free radicals (27, 28) and PrC (one of the invariable oxidative protein adjustments) which is considered as an early marker of protein oxidative stress-related disorder levels. PrC that were produced as a result of metal-catalyzed oxidation of amino acids such as lysin, proline, arginine, and threonine remainders as a result of the production of carbonylated forms. Thus, the PrC levels are considered as a protein oxidation marker. Also, GSH (a portion of the natural antioxidant system that neutralizes the oxidative stress caused by free radicals) content in newborn mice significantly decreased (28). These findings were in agreement with the other reports (29). There is some evidence which demonstrated that oxidative stress can play an important role in altering lipids, (has shown teratogenic effects of some drugs such as phenytoin and thalidomide) (30–33). Antioxidants can contribute to contrasting oxidative stress, split radical chain reactions, and prevent oxidative stress-related damage (34, 35). Our study has shown that methadone at 0.3 mg/kg could have induced apoptosis in liver tissues through transferring from mothers to neonates (mice), and this apoptosis has been shown with increasing BAX and Caspase9 expression (intrinsic markers of apoptosis) and diminishing expression of anti-apoptotic proteins Bcl2.

Methadone is a commonly prescribed treatment for opioid use disorder in pregnancy, despite limited information on the effects of passive exposure on fetal brain development. Animal studies suggested a link between perinatal methadone exposure and impaired white matter development (36). In a study, the effect of perinatal methadone exposure in rats was characterized through the evaluation of oligodendrocyte development and glial cell activation in the neonatal rat brain, it showed the increased density and percentage of oligodendrocyte precursor cells in the CC and cerebellar white matter. The highly active proliferation of oligodendrocyte precursor cells as well as decreased density and percentage of differentiated oligodendrocytes were found in the cerebellum but no differences in the cerebrum. Apoptotic activities of both differentiated oligodendrocytes and myelinating oligodendrocytes significantly increased in all regions of the cerebrum and cerebellum after methadone exposure (36).

Since melatonin plays an important role in the circadian rhythm system and also has strong antioxidant properties (37), in some clinical studies, have shown that melatonin can have a cytoprotective effect and increased efficacy of cancer chemotherapy and life expectancy. Also based on many studies, addition of melatonin to chemotherapy regime can reduce toxicity of chemotherapy and radiotherapy in patients suffering from colorectal cancer (38). In a new study in fatty liver, the effect of melatonin in apoptosis pathway (signal-regulating kinase 1) has been assayed. Although it has shown that melatonin could not have any effect on food absorption, it could reduce signs of fatty liver as weight gain and insulin desensitization and fat accumulation in the liver, significantly (39).

In a histologic study of combinational therapy of metformin and melatonin against disturbing effect of radiation in ileum and colon in 2020, it has been shown that melatonin could protect the ileum and a combination of metformin and melatonin had more effect (40). In a new study about embryo protective effect of melatonin in F1 male rats against environmental pollution of Bisphenol A, it was found that melatonin could prevent functional reproductive damage in male neonates, reduce oxidative stress, prevent testosterone reduction in mouse embryo, and prevent functional sperm defects, which did this action through its direct antioxidant and inhibition of tissue necrosis (41). In this study, we have investigated whether administration of melatonin (0.2, 0.4, and 0.6 mg/kg/day by gavages) had protective effects against oxidative stress and apoptosis induced by placental transfer of methadone in mice, which is in parallel with similar previous studies which have shown a cytoprotective effect of melatonin (34–41).

Our findings clearly showed that embryo cytoprotective effect of melatonin is dose-dependent and administration of melatonin at a high dose had the most significant effect on the diminution of the LPO level. Moreover, it caused a significant increase in GSH and decrease of PrC contents in all tissues. Although we have seen the anti-apoptotic effect for melatonin in a dose-dependent manner, our data were not significant and needed more accurate studies. Numerous studies have demonstrated the protective effect of melatonin against oxidative stress similar to our study (16, 37, 38).

## 5. Conclusion

In conclusion, our results have shown that melatonin has significant antioxidant effects in preventing oxidative damage, induced by the transfer of trans placental methadone in mice via inhibiting oxidative stress, which may be considered for more studies in clinical trials.

##  Data availability

Data were available at the following link: https://drive.google.com/drive/folders/1MCUdw0X7y6rVOrYPIAiFNEqIReOxu3EO?usp=share_link.

##  Author contributions 

M.A: Executive of experimental process, F.N: Second supervisor and analysis of data, A.D: Designer of animal reproduction study, F.Sh: Designer of oxidative stress study, R.A: Supervisor and designer of study and report writing and editing.

##  Conflict of Interest

The authors declare that there is no conflict of interest.
